# Structural Behaviour of Solid Solutions in the NdAlO3-SrTiO3 System

**DOI:** 10.1186/s11671-017-1937-8

**Published:** 2017-02-23

**Authors:** Natalia Ohon, Roman Stepchuk, Kostiantyn Blazhivskyi, Leonid Vasylechko

**Affiliations:** Lviv Polytechnic National University, 12 Bandera Street, Lviv, 79013 Ukraine

**Keywords:** Perovskite aluminates and titanates, Crystal structure, Solid solution, Phase transition

## Abstract

Single-phase mixed aluminates-titanates Nd_1-*x*_Sr_*x*_Al_1-*x*_Ti_*x*_O_3_ (*x* = 0.3 ÷ 0.9) were prepared from stoichiometric amounts of constituent oxides Nd_2_O_3_, Al_2_O_3_, TiO_2_ and strontium carbonate SrCO_3_ by solid-state reaction technique in air at 1773 K. Crystal structure parameters of Nd_1-*x*_Sr_*x*_Al_1-*x*_Ti_*x*_O_3_ were refined by full-profile Rietveld refinement in space groups *R* $\overline{3}$ *c* (*x* = 0.3, 0.5, 0.7 and 0.8) and *Pm* $\overline{3}$ *m* (*x* = 0.9). Comparison of the obtained structural parameters with the literature data for the end members of the system NdAlO_3_ and SrTiO_3_ revealed formation of two kinds of solid solutions Nd_1-x_Sr_x_Al_1-x_Ti_x_O_3_ with the cubic and rhombohedral perovskite structure. Morphotropic rhombohedral-to-cubic phase transition in Nd_1-x_Sr_x_Al_1-x_Ti_x_O_3_ series occurs at *x* = 0.84. Based on the results obtained as well as the literature data for the parent compounds, the tentative phase diagram of the NdAlO_3_–SrTiO_3_ pseudo-binary system have been constructed.

## Background

Mixed aluminates-titanates with perovskite structure formed in the *R*AlO_3_–*A*TiO_3_ pseudo-binary systems (*R* = rare earths, *A* = Sr, Ca) are prospective functional materials. In conjunction with alkaline-earth titanates, rare earth aluminates reveal excellent temperature-stable high-*Q* microwave dielectric properties and are widely used as radio-frequency ceramics in modern electronic devices ([1–6] and references herein). The highest *Q*-values among *R*AlO_3_- and *A*TiO_3_-based microwave ceramics were reported for LaAlO_3_–SrTiO_3_ system, which exhibits solid solubility across the entire compositional range. It was shown that dielectric properties of mixed aluminates-titanates ceramics do not significantly depend on the nature of the rare earth and the value of resonant frequency (*t*_f_) can be tuned by changing the concentration of solid solution. Thus potentially useful ceramics with temperature-stable relative permittivity can be obtained in other *R*AlO_3_–*A*TiO_3_ perovskite series.

The interest to the *R*AlO_3_–SrTiO_3_ systems has been increased considerably during the last decade after discovering of the intrigue phenomena of two-dimensional electron gas at the interface between two insulators LaAlO_3_ and SrTiO_3_ [7]. The interface effects occurred in the *R*AlO_3_–SrTiO_3_ perovskite systems are in the focus of active research in the field of tunable metal-insulator transition, 2D superconductivity, coexistence of superconductivity and ferromagnetism, etc. [8–11].

The aim of the present work is the study of the phase and structural behaviour of the mixed aluminates-titanates formed in the NdAlO_3_–SrTiO_3_ pseudo-binary system. At room temperature, the end members of the system—NdAlO_3_ and SrTiO_3_—adopt different variants of the perovskite structure: rhombohedral *R* $\overline{3}$ *c* and cubic *Pm* $\overline{3}$ *m*, respectively. Rhombohedral NdAlO_3_ transforms into the cubic perovskite structure near 2100 K ([6] and references herein), whereas strontium titanate SrTiO_3_ undergoes a low-temperature (LT) phase transition from the cubic to tetragonal *I*4*/mcm* perovskite structure below 105 K [12, 13]. Owing to the abovementioned peculiarities of NdAlO_3_ and SrTiO_3_ crystal structures, complex phase and structural behaviour is expected in the mixed neodymium-strontium aluminate-titanate system.

## Methods

Mixed aluminates-titanates of nominal compositions Nd_1−*x*_Sr_*x*_Al_1−*x*_Ti_*x*_O_3_ (*x* = 0.3, 0.5, 0.7, 0.8, 0.9) were prepared by solid-state reaction technique. Stoichiometric amounts of the constituent oxides Nd_2_O_3_, Al_2_O_3_, TiO_2_ and strontium carbonate SrCO_3_ were ball-milled in ethanol for 5 h, dried, pressed into pellets and annealed in air at 1673 K for 9 h. After cooling the product, it was regrinded and repeatedly annealed at 1773 K for 9 h. X-ray powder diffraction technique (Huber imaging plate Guinier camera G670, Cu K_α1_ radiation, λ = 1.54056 Å) was used for the phase and structural characterization of the samples at room temperature. All crystallographic calculations including full-profile Rietveld refinement were performed by using WinCSD program package [14].

## Results and Discussion

**Fig. 1:**
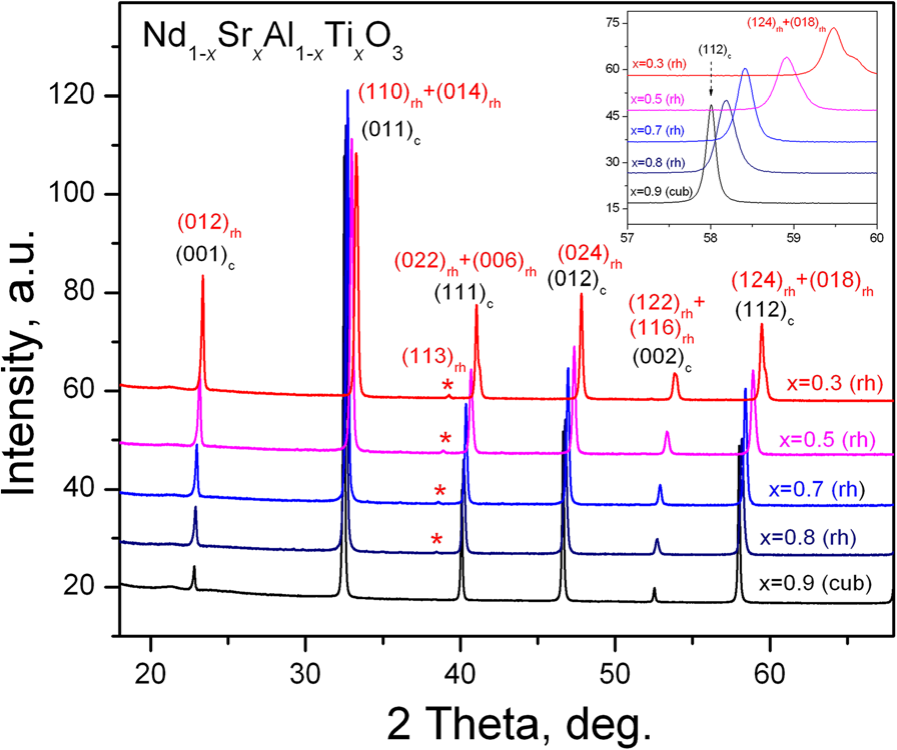
Experimental XRD patterns of the Nd_1-x_Sr_x_Al_1-x_Ti_x_O_3_ series. *Stars* indicate a superstructure (113) reflection. *Inset* shows enlarger part of patterns

Analysis of X-ray diffraction (XRD) data collected at room temperature (RT) showed that all samples synthesized adopt pure perovskite structure. No traces of foreign phases were detected (Fig. 1). Close examination of diffraction maxima revealed detectable rhombohedral deformation of the Nd_1−*x*_Sr_*x*_Al_1−*x*_Ti_*x*_O_3_ samples with *x* = 0.3 and 0.5, whereas no visible reflections splitting or deformation was observed for the specimens with higher *x* values (Fig. 1, inset). However, a presence of minor superstructure (113) reflection, which is indicative for rhombohedral distortion of perovskite structure, testifies that the rhombohedral structure in Nd_1−*x*_Sr_*x*_Al_1−*x*_Ti_*x*_O_3_ series persists at least up to *x* = 0.8.

**Fig. 2:**
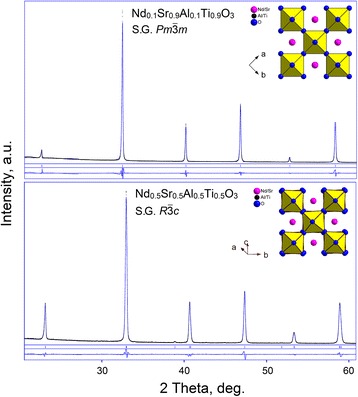
Graphical results of Rietveld refinement of Nd_0.1_Sr_0.9_Al_0.1_Ti_0.9_O_3_ and Nd_0.5_Sr_0.5_Al_0.5_Ti_0.5_O_3_ structures. The experimental X-ray powder diffraction patterns (*dots*) are shown in comparison with the calculated patterns (*blue lines*). The difference curves between measured and calculated profiles are shown below the diagrams. *Inset* shows the view of the cubic and rhombohedral structures as corner-shared Al/TiO_6_ octahedra with Nd/Sr species located between them

Full-profile Rietveld refinement of Nd_1-*x*_Sr_*x*_Al_1-*x*_Ti_*x*_O_3_ structures, performed in space groups *R* $\overline{3}$ *c* and *Pm* $\overline{3}$ *m* for the samples with *x* ≤ 0.8 and *x* = 0.9, respectively, entirely confirms suggested crystal structures of the specimens. Examples of graphical results of Rietveld refinement, showing excellent fits between experimental and calculated profiles of rhombohedral Nd_0.5_Sr_0.5_Al_0.5_Ti_0.5_O_3_ and cubic Nd_0.1_Sr_0.9_Al_0.1_Ti_0.9_O_3_ structures are presented on Fig. 2. Refined structural parameters of all synthesized Nd_1-*x*_Sr_*x*_Al_1-*x*_Ti_*x*_O_3_ samples and corresponding residuals are presented in Table 1.

**Table 1:** Unit cell parameters, coordinates and isotropic displacement parameters of atoms in Nd_1−*x*_Sr_*x*_Al_1−*x*_Ti_*x*_O_3_ structures at RT

Atoms, sites	Parameter, residuals	*x* in Nd_1−*x*_Sr_*x*_Al_1−*x*_Ti_*x*_O_3_, space group				
		0.3, *R* $\overline{3}$ *c*	0.5, *R* $\overline{3}$ *c*	0.7, *R* $\overline{3}$ *c*	0.8, *R* $\overline{3}$ *c*	0.9, *Pm* $\overline{3}$ *m*
	*a, Å*	5.3836(4)	5.4281(4)	5.4674(5)	5.4849(8)	3.8911(1)
	*c, Å*	13.1180(2)	13.251(1)	13.368(2)	13.428(3)	–
Nd/Sr, 6*c* (0, 0, ¼)	*B*_iso_ *, Å* ^*2*^	0.73(2)	0.91(1)	0.67(2)	0.83(1)	0.90(4)
Al/Ti, 6*b* (0, 0, 0)	*B*_iso_ *, Å* ^*2*^	0.54(4)	0.41(2)	0.44(3)	0.53(2)	0.48(5)
O, 18*e* (*x*, 0, ¼)	*x*	0.5395(8)	0.5358(6)	0.5277(6)	0.5213(7)	–
	*B*_iso_ *, Å* ^*2*^	1.48(8)	1.25(5)	1.72(6)	1.57(5)	1.36(11)
	*R*_*I*_	0.021	0.026	0.030	0.028	0.033
	*R*_*P*_	0.076	0.091	0.083	0.086	0.092

**Fig. 3:**
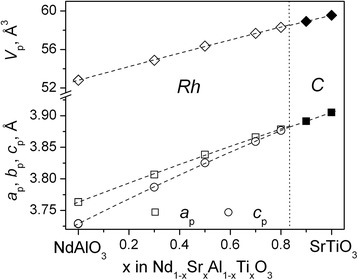
Unit cell dimensions of Nd_1-x_Sr_x_Al_1-x_Ti_x_O_3_ series. The rhombohedral lattice parameters are normalized to the perovskite cell as follow: *a*_*p*_ *= a*_*r*_/√2,*c*_*p*_ *= c*_*r*_/√12,*V*_*p*_ *= V*_*r*_ */6*. The *dotted line* marks the phase boundary between the *Rh* and the *C* phases

Concentration dependencies of the obtained lattice parameters and unit cell volumes of Nd_1-*x*_Sr_*x*_Al_1-*x*_Ti_*x*_O_3_ series in comparison with the literature data for NdAlO_3_ [6] and SrTiO_3_ [12] (Fig. 3) prove a formation of two kinds of solid solutions in the NdAlO_3_–SrTiO_3_ pseudo-binary system. Simultaneous aliovalent substitution of Sr^2+^ and Ti^4+^ species for Nd^3+^ and Al^3+^ sites reduces rhombohedral deformation in Nd_1-*x*_Sr_*x*_Al_1-*x*_Ti_*x*_O_3_ series and led to morphotropic phase transition to the cubic perovskite structure at *x* = 0.84 (Fig. 3). In the related systems LaAlO_3_–SrTiO_3_ and PrAlO_3_–SrTiO_3_, the phase boundary between two perovskite structures takes place above *x* = 0.8 and at *x* = 0.88, respectively [15, 16].

**Fig. 4:**
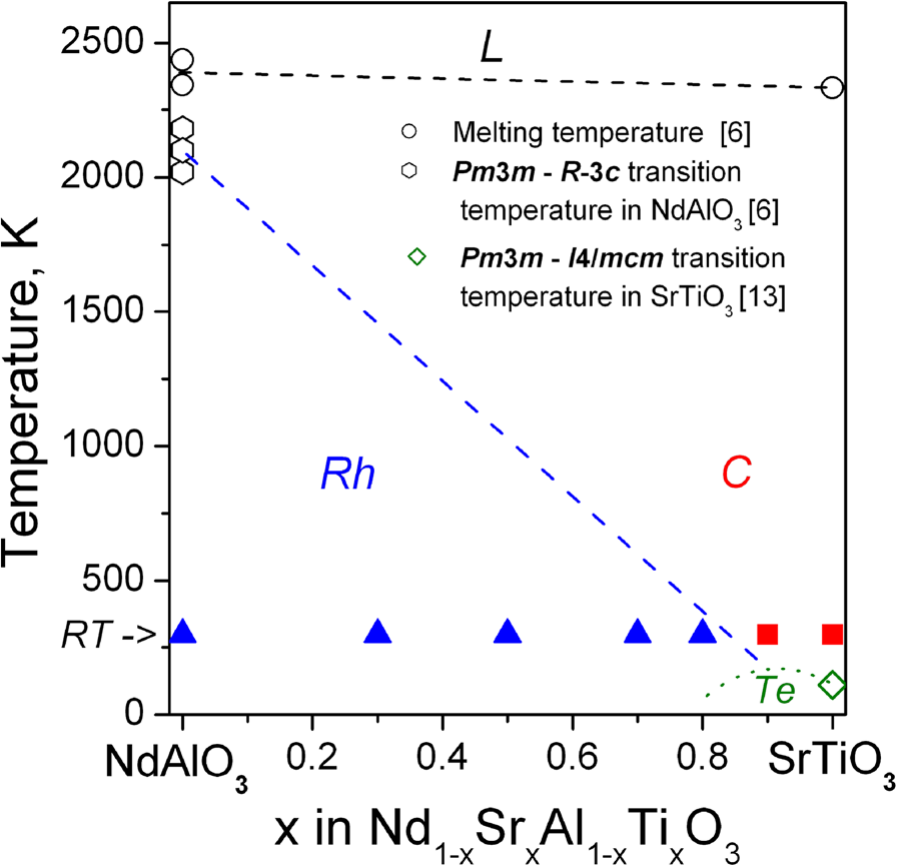
Tentative phase diagram of the PrAlO_3_–SrTiO_3_ pseudo-binary system. The letters *L*, *C*, *Rh* and *Te* designate liquid, cubic, rhombohedral and tetragonal phase fields, respectively. The solid symbols designate the rhombohedral (*triangles*) and cubic (*squares*) perovskite structures experimentally observed in Nd_1-x_Sr_x_Al_1-x_Ti_x_O_3_ series at RT

A decreasing structural deformation in Nd_1−*x*_Sr_*x*_Al_1−*x*_Ti_*x*_O_3_ series as a consequence of increasing Goldschmidt tolerance factor with increasing *x* should significantly effect on the temperature of structural phase transition *R* $\overline{3}$ *c − Pm* $\overline{3}$ *m*, which occurs in NdAlO_3_ at about 2100 K [6]. According to structural phase diagram of the related LaAlO_3_–SrTiO_3_ system [15], the temperature of rhombohedra-to-cubic transition decreases almost linearly from 850 K in “pure” LaAlO_3_ to 350 K in the sample with nominal composition La_0.2_Sr_0.8_Al_0.2_Ti_0.8_O_3_. Our recent in situ X-ray synchrotron powder diffraction investigations of the PrAlO_3_–SrTiO_3_ series [16] showed that the *R* $\overline{3}$ *c − Pm* $\overline{3}$ *m* transition temperature decreases gradually from 1770 K in PrAlO_3_ to 930 K in Pr_0.5_Sr_0.5_Al_0.5_Ti_0.5_O_3_ sample. Similar structure and phase behaviour at the elevated temperatures are also expected in the studied NdAlO_3_–SrTiO_3_ system, tentative phase diagram of which is shown on Fig. 4. However, extrapolation of the cubic phase boundary from high-temperature region to the higher SrTiO_3_ concentrations would be speculative because of the different low-temperature structures of the parent compounds NdAlO_3_ and SrTiO_3_ (*R* $\overline{3}$ *c* and *I*4/*mcm*, respectively). Evidently, phase boundary between the *R* $\overline{3}$ *c* and *I*4/*mcm* structural modifications of Nd_1-*x*_Sr_*x*_Al_1-*x*_Ti_*x*_O_3_ solid solution has to be present in the SrTiO_3_-rich part of the phase diagram at low temperatures (Fig. 4). In addition, appearance of intermediate orthorhombic phase between rhombohedral *R* $\overline{3}$ *c* and tetragonal *I*4/*mcm* phase fields, as it occurs in the related PrAlO_3_–SrTiO_3_ [16] and NdAlO_3_–CeAlO_3_ [17] systems, could not be neglected.

Comprehensive analysis of *A*/*B*-cation substitution on the antiferrodistortive phase transition *Pm* $\overline{3}$ *m* − *I*4/*mcm* in SrTiO_3_ recently performed in [18] revealed that transition temperature increases in nonlinear manner with decreasing tolerance factor, depending on substituent concentration. Based on this observation, one can predict that SrTiO_3_-richest samples in the NdAlO_3_–SrTiO_3_ system will transform to the tetragonal *I*4/*mcm* structure at the temperatures higher than the pure SrTiO_3_ (105 K). To shad light on the low-temperature phase behaviour in the NdAlO_3_–SrTiO_3_ system, thorough structural, calorimetric and spectroscopic investigations are required.

## Conclusions

Continuous solid solution Nd_1-x_Sr_x_Al_1-x_Ti_x_O_3_ with perovskite structure is formed in the NdAlO_3_–SrTiO_3_ pseudo-binary system at 1773 K. Comparison of the obtained structural parameters with corresponding data for the parent compounds NdAlO_3_ and SrTiO_3_ proves a decrease of perovskite structure deformation as a consequence of increasing Goldschmidt tolerance factor with increasing *x* in Nd_1-x_Sr_x_Al_1-x_Ti_x_O_3_ series. As a result, concentration-induced phase transition from a rhombohedral *R* $\overline{3}$ *c* to the cubic perovskite structure occurs in the Nd_1-x_Sr_x_Al_1-x_Ti_x_O_3_ system at *x* = 0.84. Experimental X-ray powder diffraction patterns and crystal structure parameters of rhombohedral Nd_0.7_Sr_0.3_Al_0.7_Ti_0.3_O_3_ and cubic Nd_0.1_Sr_0.9_Al_0.1_Ti_0.9_O_3_ phases are published by the International Centre of Diffraction Data (ICDD) in the last release of the Powder Diffraction Files (PDF cards NN 00-066-0395 and 00-066-0396, respectively) [19].
